# Tribo-mechanical properties evaluation of HA/TiO_2_/CNT nanocomposite

**DOI:** 10.1038/s41598-021-81187-7

**Published:** 2021-01-21

**Authors:** Erfan Zalnezhad, F. Musharavati, Tianyi Chen, Fadi Jaber, Kaan Uzun, Muhammad E. H. Chowdhury, Amith Khandakar, Junxing Liu, S. Bae

**Affiliations:** 1 Department of Biomedical Engineering and Chemical Engineering, University of Texas at San Antonio, San Antonio, TX USA; 2 Mechanical and Industrial Engineering Department, College of Engineering, Qatar University, 2713 Doha, Qatar; 3 Department of Mechanical Convergence Engineering, Hanyang University, Seoul, South Korea; 4 Department of Biomedical Engineering, Ajman University, Ajman, United Arab Emirates; 5 Mechatronics R&D Center, Samsung Electronics Co., Ltd., Hwaseong, South Korea; 6 Department of Electrical Engineering, College of Engineering, Qatar University, 2713 Doha, Qatar; 7 Department of Architectural Engineering, Hanyang University, Seoul, 04763 South Korea

**Keywords:** Medical research, Engineering, Materials science

## Abstract

In this study, a combination of reverse microemulsion and hydrothermal techniques were used to synthesize HA. A hydrothermal method was used to synthesize HA/TiO_2_/CNT nanocomposite powders. Cold and hot isostatic pressing techniques were used to fabricate tablet-shaped samples. To investigate the biocompatibility and tribo-mechanical properties of HA/TiO_2_ and HA/TiO_2_/CNTs, four samples were prepared with different percentages of CNTs, namely, HA/TiO_2_ (S0), HA/TiO_2_/CNT (S1.0), HA/TiO_2_/CNT (S2.0), and HA/TiO_2_/CNT (S3.0). The microstructure and morphology of the HA/TiO_2_/CNTs were characterized by transmission electron microscopy, scanning electron microscopy, energy-dispersive X-ray spectroscopy, and X-ray diffraction. Hardness test results show that S3.0 displayed the highest surface hardness (285 HV) compared to other samples. The wear rate of HA/TiO_2_/CNT with the highest CNT content showed a decrease compared with those of the other samples. The results from nanoindentation tests showed that Young’s modulus of the S3.0 sample was 58.1% greater than that of the S0 sample. Furthermore, the human MDA-MB-231 cell line demonstrated good binding to the surface of the samples in the in-vitro biocompatibility evaluation of the HA/TiO_2_/CNT composites.

## Introduction

Bioceramics sintered at high temperature have good mechanical strength and hardness, are difficult to dissolve in the body, are resistant to oxidization and corrosion deterioration, have thermal stability, are easy to heat to combat disinfection, and demonstrate proper wear resistance^1–4^. Ceramics can be fabricated into various shapes and sizes, such as granular, cylindrical, and tubular forms, and can be compact or porous. They can also be used as bone screws, bone splints, collar bones, or skull components^5^. Furthermore, they are easily colored; for example, ceramics used in teeth can be color-matched to the color of natural teeth. Also, bioceramics can be applied to artificial hearts, artificial joints, and artificial tooth roots and can be used as bone fillings, bone replacements, and bonding materials^5–7^.

Bioinert ceramics mainly refer to materials with the chemical properties of stable and good biocompatibility. The structure of these ceramic materials is relatively stable, and the material shows high mechanical strength, wear resistance, and chemical stability^8,9^. Al, Mg, Ti, and Zr oxides are the most widely used metal oxides in biomedical applications^10–12^.

Biologically active ceramics include surface-bioactive ceramics and bioabsorbable ceramics, also known as biodegradable ceramics. Bio-surface-active ceramics usually contain hydroxyl groups and can be made porous for biological tissue to grow on and firmly bond with the ceramic surface^13^. Bio-absorbent ceramics are characterized by partial absorption or complete absorption in vivo and can induce new bone growth. Examples of biologically active ceramics are bioactive glass, hydroxyapatite ceramics, and tricalcium phosphate ceramics^14–16^.

Hydroxyapatite (HA) is a surface-active material with the chemical formula Ca_10_(PO_4_)_6_(OH)_2_. Because the main component of hard human tissue (tooth and bone) is hydroxyapatite limestone, HA is also called hydroxyapatite ceramic artificial bone. It is a ceramic material with high bioactivity and biocompatibility. It is non-toxic, non-carcinogenic, and biodegradable, and it has the ability to directly bond with the bone. However, the main disadvantage of HA is its poor mechanical properties, including low fracture toughness and brittleness, which represent shortcomings for its wider clinical application to medicine. These limitations have encouraged the study of HA in composite materials^17–21^.

Various studies on dense HA ceramics have been conducted to enhance the mechanical properties of HA. In recent years, greater focus has been directed toward the study of porous hydroxyapatite ceramics. Porous calcium phosphate implants mimic the structure of the bone matrix and have osteoinductive properties, which can provide the structure and channels for the growth of new bone tissue; thus, the tissue response to a porous calcium phosphate implant is much better than that of dense ceramics^22^. The success of a surgical implant depends not only on the bone-implant combination but also on the sterility of the surrounding surface of the implant, preventing bacterial infection associated with the material. As such, there is a demand for a material that can prevent bacterial infection. Studies have shown that the calcium ions in the HA lattice can be easily replaced by other metal ions, and such a replacement may result in improved bioactivity and promote bone binding and even antibacterial activity^23,24^.

Carbon nanotubes (CNTs) are known as super-nanomaterial. The unique structure, excellent performance, and broad prospects for scientific and technological applications of CNTs have received widespread attention^22,25–27^. CNTs possess excellent mechanical properties and can withstand the high strain. Reportedly, CNTs exhibit a significant elastic modulus greater than 1 TPa and tensile strength of 30 GPa^28^. Therefore, CNTs are considered as an ideal one-dimensional nanocrystalline toughening material.

Zhan et al. added 10% single-walled CNTs to a nano-A1_2_O_3_ sample. The hardness of the composites was 16.1 GPa after 3 min of discharge plasma sintering at 1500 °C. Ning et al. added 5% multi-walled CNTs to SiO_2_, and the composite bending strength and fracture toughness increased by 88% and 146%, respectively^29–33^. Kealley et al. reinforced HA with CNTs for mechanical enhancement. Zanello et al. also showed that osteoblasts can grow and proliferate on CNTs^34–36^. Pandey et al. studied the Bacterial and Tribological properties of Ti-4 V coated by HA/CNT/Ceria/Ag composite. The hardness, fracture toughness, elastic modulus, and wear resistance of the coated Ti-4v improved significantly. They also found that the HA/CNT/Ceria/Ag composite coating was cytocompatible^37^. In another study, Pandey et al. investigated the tribological properties of HA/CeO_2_/Ag composite coating onto titanium alloy. They Observed that the wear resistance of the coated sample increased by 89% (by fretting) and 13% (by scratch), by the addition of CeO_2_ and Ag reinforcement^38^. The Effects of boron nitride nanoplate on the mechanical properties of the hydroxyapatite composites prepared by spark plasma sintering investigated by Aguirre et al. The boron nitride nanoplate reinforced HA composites showed enhancement of 2.3 MPa and 79.79 MPa in fracture toughness and flexural strength of HA compared to the other researches (1.0 MPa). They claimed that regardless of the Weibull Distribution which indicated a sacrifice in mechanical reliability, all the composites synthesized in their study exhibited a low possibility of failure and a safety factor of ~ 5.6 was achieved^39^. Nezhad et al. studied the effect of titanium and carbon nanotubes on nano/micromechanical properties, wetability and biocompatibility of hydroxyapatite. They discovered that addition of 1% titanium nanotubes and 2% carbon nanotubes to the hydroxyapatite improved the nanoscratch and hydrophilicity, and reduced the cytotoxicity of hydroxyapatite composite compared with pure hydroxyapatite^40^. Awasthi et al. investigated the tribological properties of Ti-4v coated by HA/CNT using the electrophoretic deposition technique. First, they created channels on the surface of the titanium alloy using the abrasive water jet technique. Second, HA/CNT was deposited onto the titanium alloy substrate. The fretting wear tests conducted and the wear resistance of coated samples increased significantly due to the lower COF of the coated sample (0.22) compared to the bare substrate (0.27). They concluded that the enhancement in wear resistance of the coated sample was because of the textured channels trapping the particles released during the fretting wear test in the contact region^41^.

This research aimed to examine the biocompatibility and tribological and mechanical characteristics of HA/TiO_2_ and HA/TiO_2_/CNT (with different CNT content) nanocomposites. A hydrothermal technique was used to synthesize HA/TiO_2_/CNT nanocomposites. Hot isostatic pressure (HIP) was applied to the nanocomposites for sintering purposes. To evaluate the structure and morphology of the HA/TiO_2_/CNT nanocomposites, transmission electron microscopy (TEM), scanning electron microscopy (SEM), energy-dispersive X-ray spectroscopy (EDX), and X-ray powder diffraction (XRD) were utilized. The biocompatibility, surface hardness, and wear resistance of the composites were examined.

## Experimental details

### Material preparation

Titanium butoxide (97%) was purchased from Sigma Aldrich. Hydrochloric acid (HCl, 35%), ethylene glycol (≥ 99%), and ethyl alcohol (94.5%) were purchased from Daejung Chemicals. CaCl_2_-2H_2_O (≥ 99%) and dimethylformamide (≥ 99%) were purchased from Sigma-Aldrich. CNTs (20–30 wt%) were purchased from Nano Solution Co. Ltd., Korea.

### Sample preparation

#### *Preparation of TiO*_*2*_* powder*

**Figure 1:**
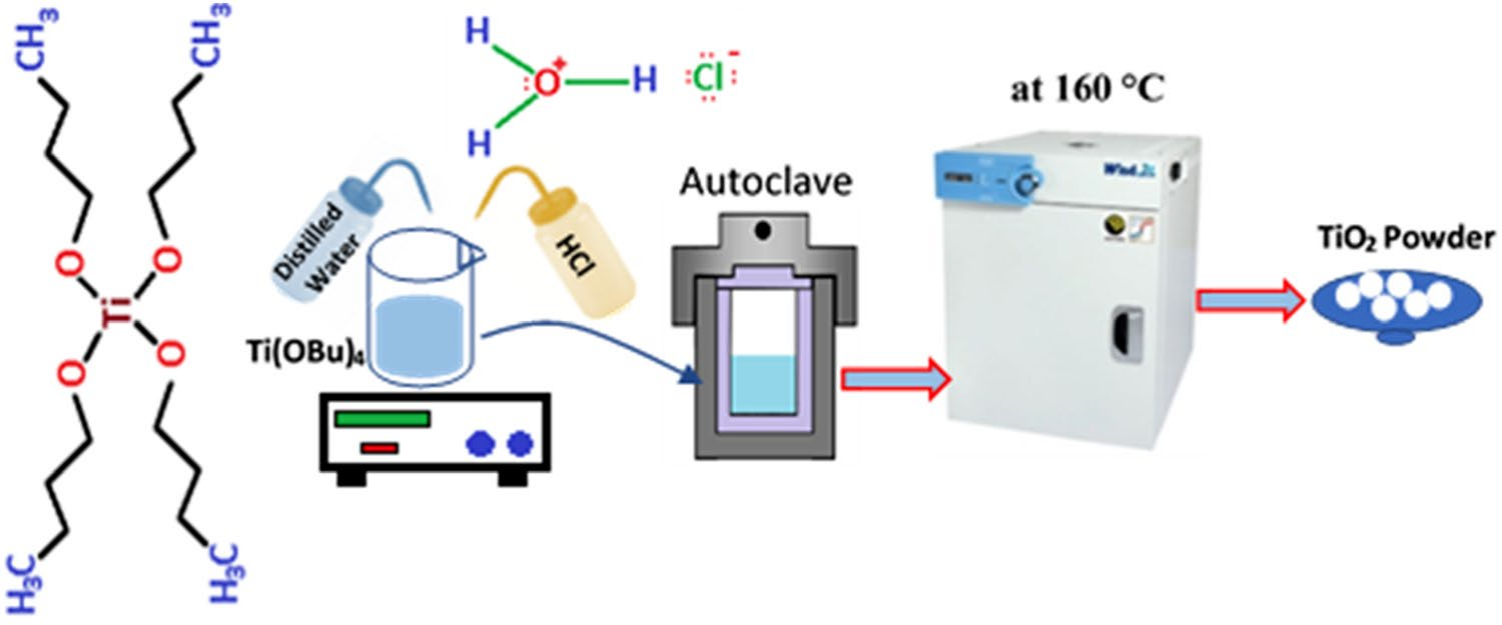
Schematic illustration of the process of synthesizing TiO_2_ nanopowder.

First, 1 ml titanium butoxide was added to 50 ml distilled water dropwise and stirred at 300 rpm for 15 min. Then, 1 ml HCl was added to the mixture dropwise and stirred for 5 min. Next, the product was transferred to a 75 ml autoclave and placed in an oven for 5 h at 160 °C. Figure 1 shows the schematic illustration of the TiO_2_ nanopowder fabrication process.

#### Preparation of HA powder

To synthesize hydroxyapatite, 2.4 mol cetrimonium bromide was added to a 10 ml 0.3 M (NH_4_)_2_HPO_4_ solution and stirred at 50 °C. The mixture was maintained at ambient temperature for 3 h to ensure that the self-assembly process and cooperative interaction were accomplished. Meanwhile, 10 ml of Ca(NO_3_)_2_ was combined with 15 ml Triton X-100, 5 ml n-butanol, and 50 ml cyclohexane, creating a mixed oil phase. Then, the cetrimonium bromide and (NH_4_)_2_HPO_4_ mixture was poured into the aforementioned reverse microemulsion under gentle and continuous stirring. Lastly, the final solution (microemulsion) was transferred into a 150 ml Teflon container and sealed tightly in a stainless steel cylinder, which was heated in an oven at 160 °C overnight.

#### Preparation of nanocomposites

**Figure 2:**
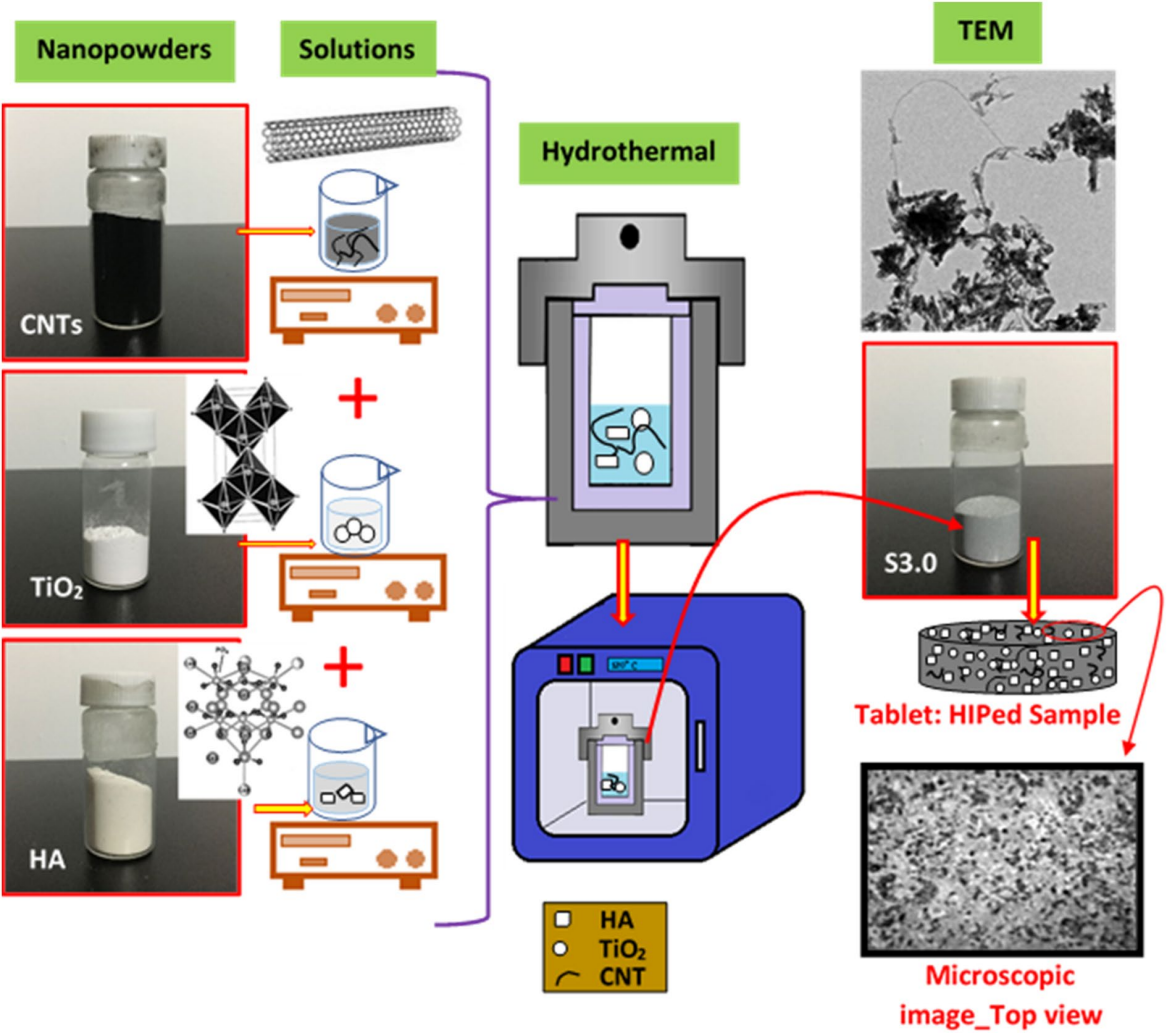
Schematic of the process of synthesizing the composite.

In this study, we prepared four different groups of sample (with 5 samples in each group) and the composition of each group is shown in Table 1. Hydrothermally synthesized HA/TiO_2_/CNTs with different compositions were HA/TiO_2_, HA/TiO_2_/CNT-1.0, HA/TiO_2_/CNT-2.0, and HA/TiO_2_/CNT-3.0, which, hereafter, are named S0, S1.0, S2.0, and S3.0, respectively. The composite powders were centrifugally cleaned with deionized water five times and then dried for 8 h in a vacuum oven at 120 °C. Figure 2 shows the schematic of the preparation process of the HA/TiO_2_/CNTs.

**Table 1:** Percentage of each material in the HA/TiO_2_/CNT composites.

Name	HA%	TiO_2_%	CNT%
S0	90	10	0
S1.0	89.6	9.4	1
S2.0	88.6	9.4	2
S3.0	87.6	9.4	3

#### Composite tablet preparations

The CIP process was performed before the HIP process to produce a relatively dense tablet. The composite powder was uniaxially compressed into a disc using a 10 mm diameter tungsten carbide (WC) mold at 250 MPa. HIP was conducted at 1150 °C for 1 h in an argon gas atmosphere with high purity at 160 MPa. Both heating and cooling rates were maintained under 5 °C per min. The heat-treated specimens were 3 mm thick and 10 mm in diameter. Before the mechanical performance test, the HIPed specimens were mounted with epoxy resin. The surface of the specimens was polished with SiC paper of 600–2000 grit. Final polish was conducted with 0.5, 3, and 9 µm (particle size) polishing suspension to achieve a smooth surface with uniform roughness all over the specimens.

#### Evaluation of microhardness

Samples were tested for hardness with a Vickers microhardness tester (Shimadzu HMV-2000, Shimadzu Corporation, Japan). Loading was terminated once the depth of penetration reached its maximum level; thereafter, the force was retained steady for 5 s. Micro-indentation tests were carried out on four specimens, and 10 indentations were performed on each specimen with a force of 1.961 N. Microscopic images were provided to characterize the impressions after indentation.

#### Nanoindentation

To characterize the nanomechanical properties of the nanocomposite, such as modulus of elasticity, a Hysitron nanoindenter apparatus was used. A standard fused quartz substrate was used for calibration purposes. Nanoindentation was carried out with 10 s loading and unloading as well as a holding time of 3 s at the maximum load. The modulus of elasticity was calculated using the Oliver–Pharr method^31^.

#### Tribology test

The wear resistance of the samples was examined using a wear-testing machine (reciprocating) with a ball made of stainless steel (4 mm diameter) as a normal load. The balls and tablets were washed in distilled water and acetone before the start of the test. A normal load of 10 N was applied to the tablet under reciprocating conditions. The friction coefficient (µ_k_) was calculated by dividing friction force F by normal force N. The rate of wear was evaluated using a high-precision (0.0001 mg) weight balance (METTER TOLEDO, Switzerland).

### Characterization

Microstructural and morphological characterizations of the composite powders (HA/TiO_2_/CNTs) were carried out through high-resolution SEM (FEI NOVA Nano 450) and TEM. The crystalline phase of the specimens was analyzed by an XRD powder (PANalytical’s Empyrean) armed with a monochromated Cu Kα radiation source (k = 1.54056 Å). EDX analysis was conducted to analyze the elemental composition of the specimens by using an EDX system (S-2380N, Hitachi) connected to the SEM.

### In vitro biocompatibility tests

To visualize the feasible cells, the human MDA-MB-231 cell line, which exhibits effective green fluorescent protein (GFP), was utilized. Cells were grown with 10% fetal bovine serum (FBS) and 1% pen-strep (PS) antibiotics in T-25 culture flasks, using Dulbecco's adapted Eagle medium (DMEM). Cells were kept in a culture incubator until reaching 80% confluency under an atmosphere of 5% CO_2_ at 37 °C. Next, cells were trypsinized, centrifuged, and re-suspended in 1 mL of DMEM (10% FBS, 1% PS). Then, 100 L of cell suspension was applied to the surfaces of HA, HA/TiO_2_, and HA/TiO_2_/CNT. The specimens remained intact for 20 min; thereafter, 5 mL of DMEM media (1% PS, 10% FBS) was applied to ensure that the cells were immersed in the culture medium. The specimens were then placed into the culture incubator for the next 3 days. Then, through the addition of paraformaldehyde, the cells were fixed on the third day. Fluorescence imaging was carried out by using an inverted microscope (Nikon, Japan) with a FITC-LP01 filter array (Semrock Inc., USA) to visualize the cell morphology.

## Results and discussion

### Biocompatibility test

**Figure 3:**
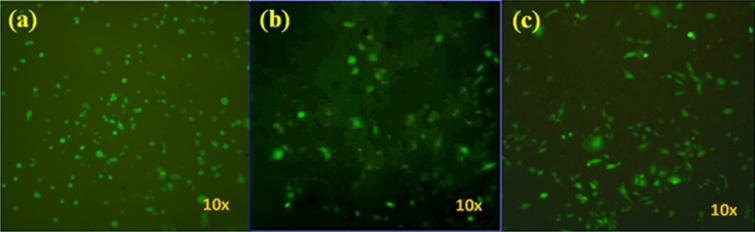
Growth of MDA-MB-231 cells on the surface of (**a**) HA, (**b**) S0, and (**c**) S3.0 nanocomposites.

Figure 3 shows the biocompatibility test on the surfaces of the HA, S0, and S3.0 nanocomposites. The results of the biocompatibility test for the pure HA and HA/TiO_2_ composites without CNT are shown in Fig. 3a,b, respectively. As shown in Fig. 3c, the HA/TiO_2_/CNT nanocomposite allowed cell proliferation and attachment, as evidenced by the fluorescent GFP signal generated from the MDA-MB-231 cells. Furthermore, the observation of cell diffusion on the surface of the specimen indicates a typical epithelial morphology, indicating high surface adhesion and nanocomposite biocompatibility. These findings suggest that the cells are efficiently attached and proliferated on the surfaces of the S3.0, HA, and S0 specimens.

### Morphology and structure

**Figure 4:**
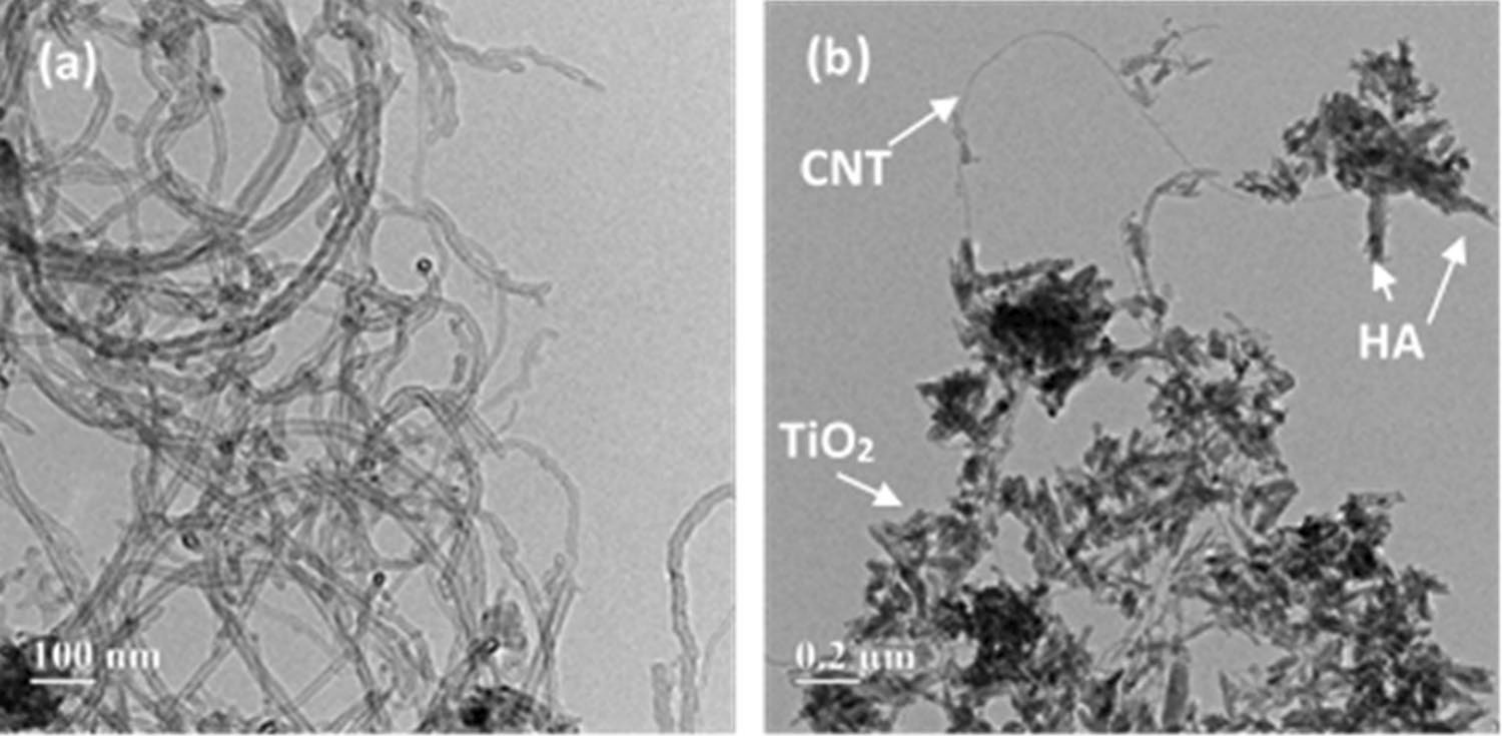
TEM images of (**a**) CNTs and (**b**) S3.0 sample.

**Figure 5:**
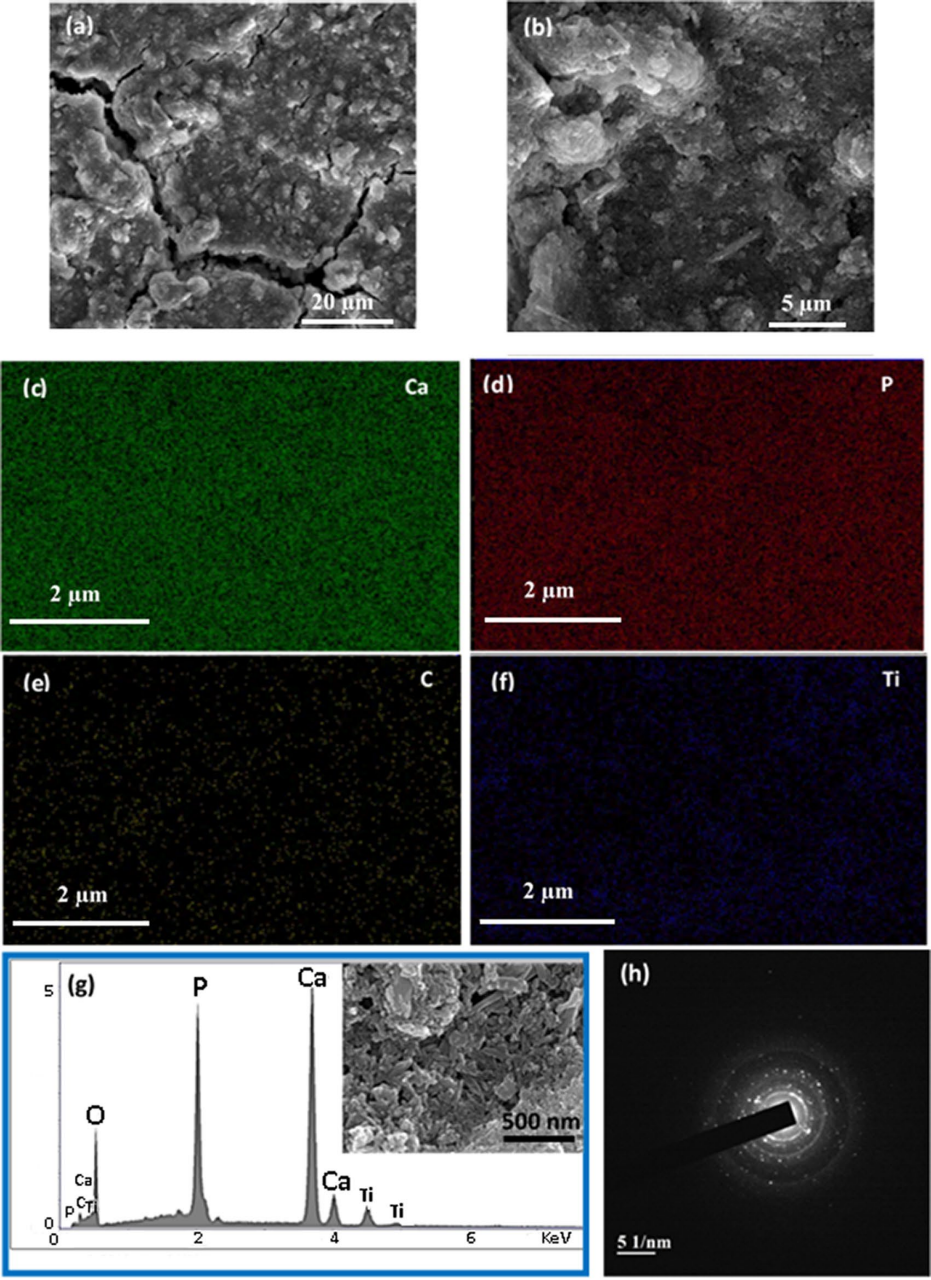
(**a**,**b**) SEM views at 20 µm and 5 µm magnifications, respectively; (**c**–**f**) EDX elemental mapping; (**g**) elemental composition of nanocomposite (the selected area is embedded in the EDX); and (**h**) SAED. Pictures show the S3.0 sample.

Figure 4a,b show the TEM images of the CNTs and S3.0 samples. As can be seen from the image, CNTs have a diameter of approximately 20 nm and a length of a few microns. Figure 4b indicates that TiO_2_ and HA nanoparticles are properly bounded by CNTs. To prepare the sample for the SEM and EDX tests, 2 mg of S3.0 powder was added to 4 ml distilled water and sonicated for 15 min. Then, 2 drops of the suspension were poured into a glass and dried in the oven at 70 °C for 48 h. Figure 5 shows the typical SEM micrographs of the HA/TiO_2_/CNT nanocomposites synthesized by the hydrothermal method. Figure 5a,b present the SEM of the HA/TiO_2_/CNTs at different magnifications. Higher magnification of SEM is included in Fig. 5g (inset) to better reveal the morphology and structure of the composite. CNTs can be seen along with the TiO_2_ nanoparticles. CNT, TiO_2_, and HA particles are distributed over the glass, with more HA and TiO_2_ nanoparticles shown than CNTs.

EDX analysis was performed for further investigation. Figure 5c–g present the elemental analysis and intensity of different elements, such as Ca, P, O, Ti, and C, in the S3.0 sample. To further characterize the distribution of the different elements in the nanocomposite, EDX elemental distribution mapping was performed as shown in Fig. 5g. The Ca, P, C, O, and Ti elements were evenly distributed in the HA/TiO_2_/CNT nanocomposites. Figure 5h presents the selected-area electron diffraction (SAED) analysis of the S3.0 sample; as can be seen, the composite sample exhibits a multi-crystalline structure. Table 2 shows the detailed elemental composition of the S3.0 sample. The weight percentages of C, O, P, Ca, and Ti were 1.09, 32.39, 18.76, 42.57, and 5.19, respectively.

**Table 2:** Detailed elemental composition of S1.5 sample.

Element	Weight%	Atomic%	Net int.	Error%
CK	1.09	2.34	1.60	17.56
OK	32.39	52.02	59.40	10.72
PK	18.76	15.56	221.90	3.03
CaK	42.57	27.29	289.70	2.31
TiK	5.19	2.79	25.40	5.75

### X-ray diffraction analysis

**Figure 6:**
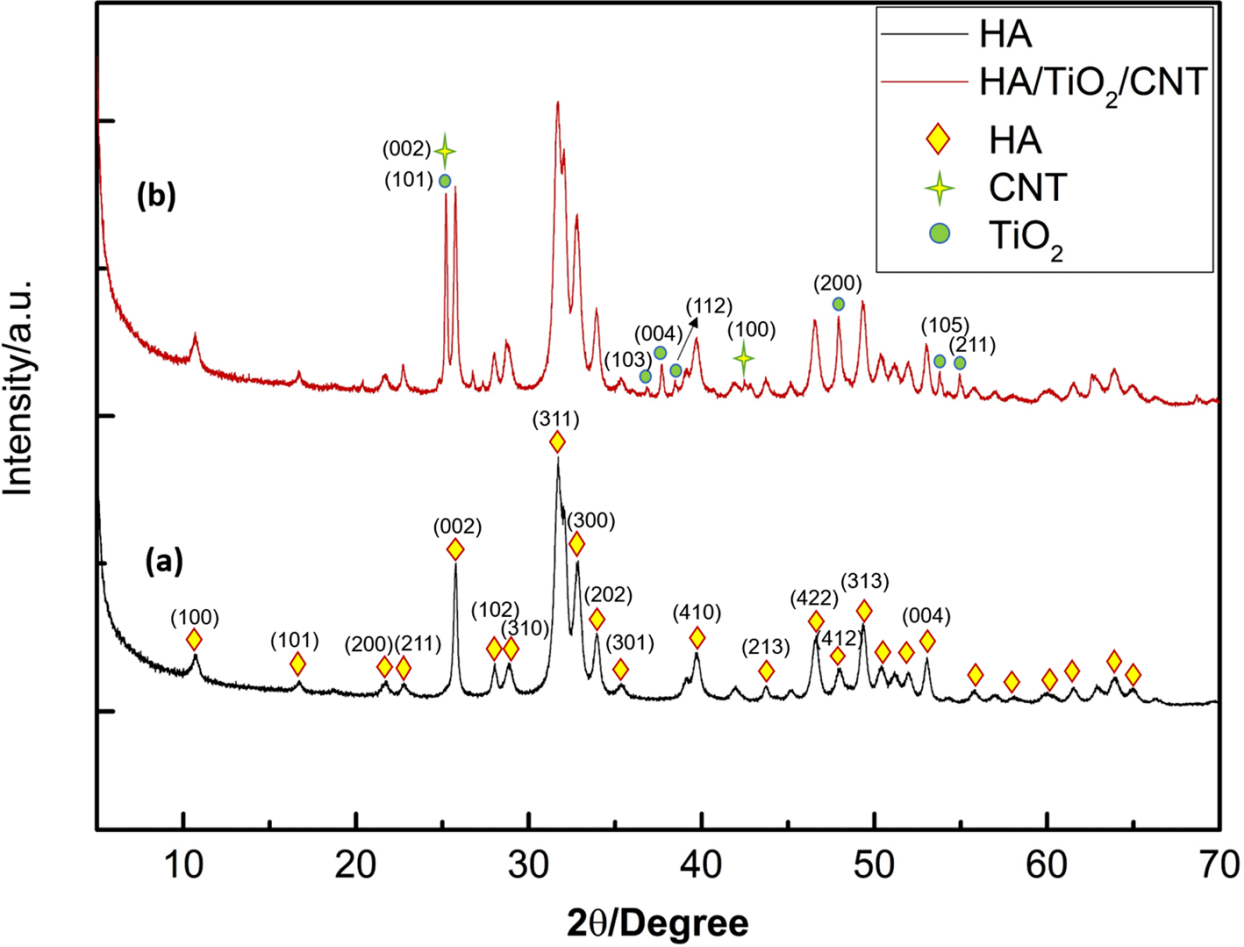
XRD spectra of the HA and S3.0 samples.

XRD patterns for the powder S0 and sintered S3.0 are shown in Fig. 6. The characteristic diffraction peaks of HA were centered at 10.8°, 16.7°, 21.5°, 22.6°, 25.8°, 28.3°, 28.9°, 31.6°, 33.2°, 34.3°, 35.6°, 39.8°, 43.6°, 46.8°, 48.2°, 49.2°, and 53.2°. The sharp and intense diffraction peaks at 2θ = 25.8°, 2θ = 31.6°, 2θ = 33.2°, 2θ = 34.1°, 2θ = 39.8°, 2θ = 46.6°, and 2θ = 49.2° of HA were attributed to the (002), (311), (300), (220), (410), (422), and (313) lattice planes, respectively (Fig. 6a). The XRD pattern of the fabricated S3.0 sample can be seen in Fig. 6b. The main peak belongs to HA (JCPDS No. 09-0432), which incorporates strong and sharp peaks as a result of the high crystallinity of the HIPed composite. According to the phase transition at HA, two main stages of tricalcium phosphate (TCP) (α-TCP and β-TCP) are considered. β-TCP (JCPDS No. 070-2065) at the two highest peaks, 2θ = 27.77 and 2θ = 31.02, does not exist in the consolidation. Meanwhile, TCP (JCPDS No. 029-0359) has no peaks at 2θ = 30.71, whereas the other peaks at 2θ = 28.8 and 2θ = 34.2 are overlapped with the peaks of HA at 2θ = 28.93 and 2θ = 34.3. Thus, we were unable to identify α-TCP or β-TCP. Such findings indicate that during the HIPed process, HA does not break down into TCP. Moreover, the characteristic diffraction peaks of the TiO_2_ at 24.9°, 37.8°, 38.9, 47.8, 53.2° corresponded to the (103), (004), (112), and (200), (105), and (211) crystal planes. Earlier research showed that in the HIPed and HA composites, in which HA only partially decomposed into TCP, the non-stoichiometric composition or trace impurities existing in the HA powders enhanced the decomposition. An exceptionally uniform composition and nanocrystalline HA-related purity stabilize the sample when it is decomposed at high temperatures^42^. In fact, the S3.0 sample contains traces of CNT and TiO_2_ after the HIP process, and their presence was confirmed by TEM, SEM, and EDS.

### Mechanical characterization

**Figure 7:**
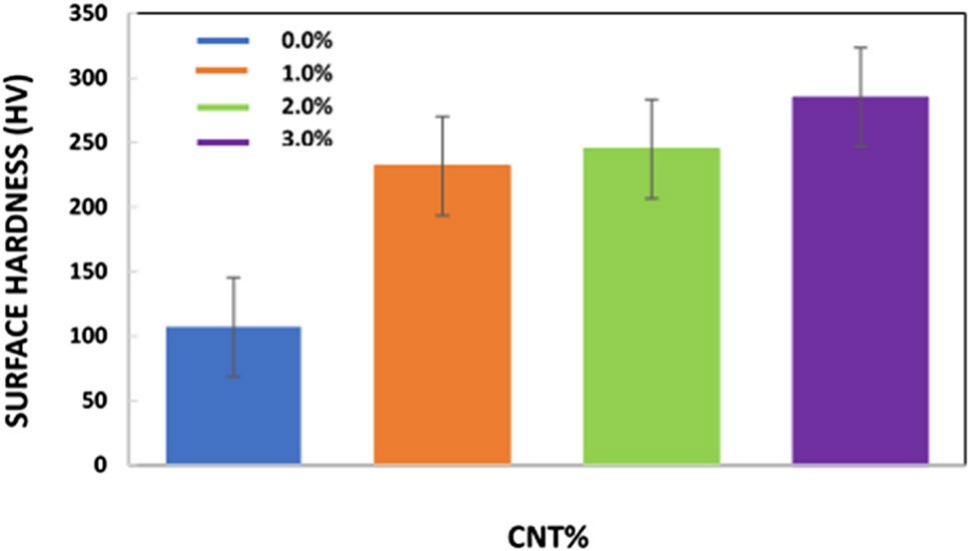
Microhardness of S0, S1.0, S2.0, and S3.0 samples.

**Figure 8:**
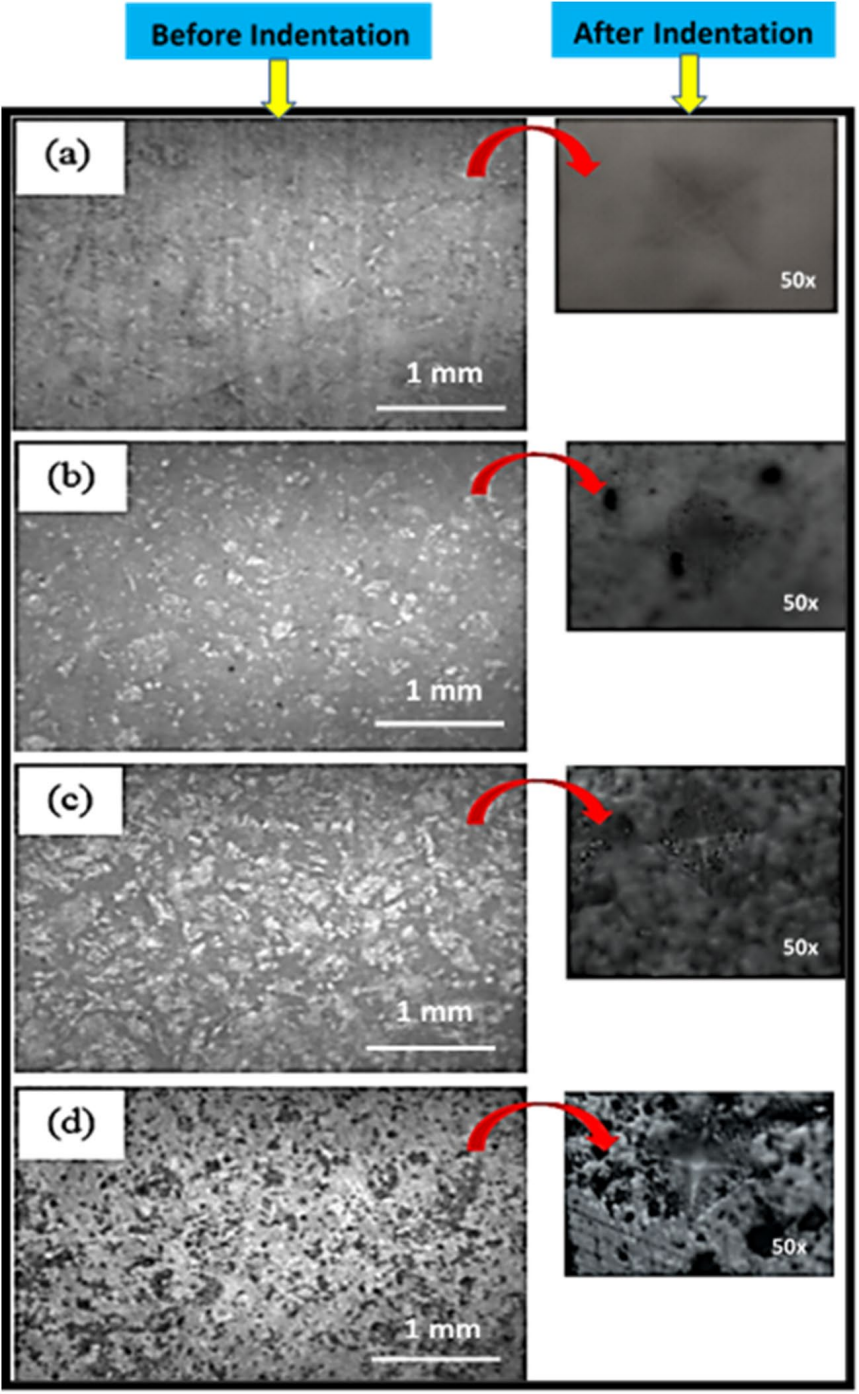
(**a**–**d**) Microscopic images of S0, S1.0, S2.0, and S3.0 samples.

Over the past few years, various studies regarding the mechanical behavior of materials have focused on micro- and nano-hardness through the nanoindentation test. In this work, the mechanical behaviors of the S0, S1.0, S2.0, and S3.0 nanocomposites were determined, as shown in Fig. 7. The surface hardness values of the composites S0, S1.0, S2.0, and S3.0 were 107, 232, 245, and 285 HV, respectively. These findings suggest that even small amounts of CNT have a notable impact on the mechanical characteristics of the bulk. The surface hardness of the S1.0, S2.0, and S3.0 samples improved by 116.8%, 129%, and 166.4%, respectively, compared with that of S0, which are significant enhancements. The hardness of the S2.0 and S3.0 sample surfaces improved by 5.6% and 22.8% compared with that of S1.0, respectively. The hardness of the S3.0 surface was 14% greater than that of S2.0, indicating that, with increasing CNT, there is a relative improvement in the surface hardness of the S3.0 nanocomposite. The mechanical properties in this study depend on the sintering method used. Hot pressing was used by Zhao et al.^43^ to determine that the hardness increased as the amount of graphene increased, and the fracture toughness improved by 75% in comparison with that of pure HA. Zhang et al.^44^ utilized the spark plasma sintering process to enhance the elastic modulus, hardness, and fracture toughness compared with those of pure HA by 31%, 43%, and 82%, respectively. For further investigation, microscopic images were captured before and after the hardness tests. Figure 8a–d present the microstructure of polished S0, S1.0, S2.0, and S3.0 composites before the hardness test. The corresponding micrographs of the tested (hardness) samples were placed in front of each image. With an increase in the CNT content from 0% to 3.0%, the size of the diamond scars due to indentation decreased because of the greater surface hardness. With an increase in the CNT content of the samples, the hardness of the CNT-reinforced hydroxyapatite composites increased significantly^45^.

The plastic deformation in the HA matrix decreases through the strengthening effect of the CNTs because CNTs have greater stiffness^46^. Higher surface hardness results in greater scratch and wear resistance that is advantageous in orthopedic implant applications^47^. The cracks propagate through the composite, reaching the CNTs and causing a deflection in the CNTs while absorbing energy; this results in the toughening of the matrix. In other words, the mechanical interlocking and proper chemical bonding of CNTs in the HA matrix requires more energy to propagate the cracks in the composite and may absorb more fracture energy^45,47^.

### Nanoindentation test

**Figure 9:**
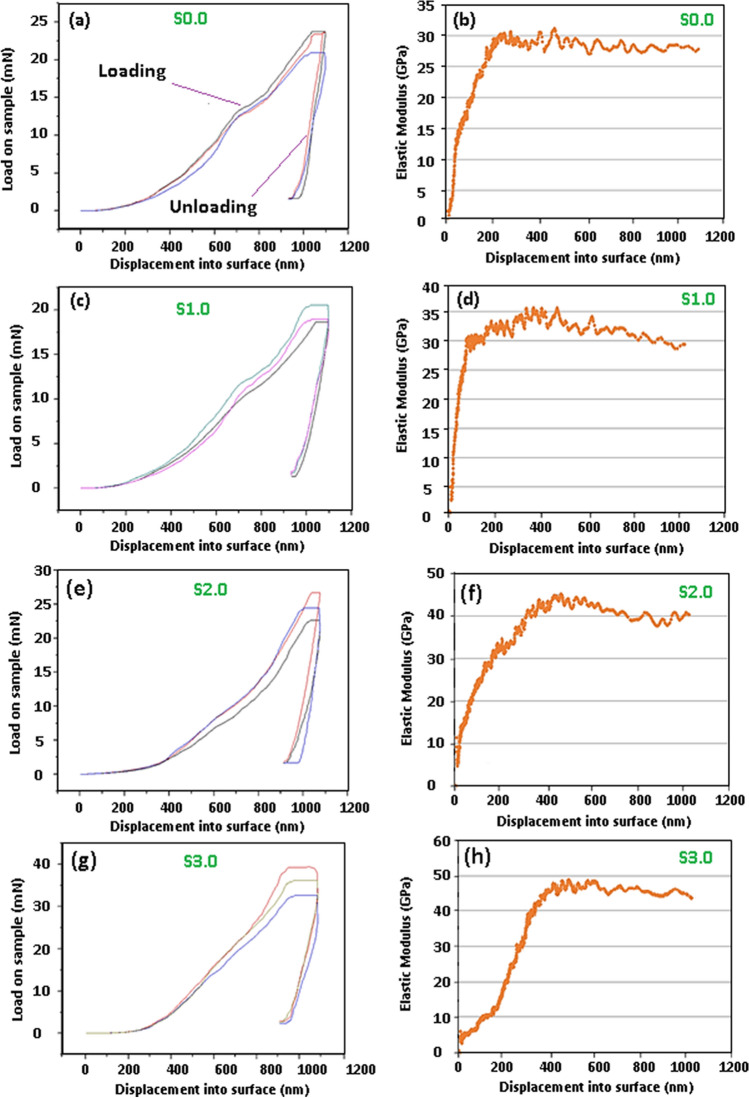
Characteristic load vs nanoindentation depth for samples S0, S1.0, S2.0, and S3.0.

In this study, nanoindentation tests were performed to evaluate the elastic moduli of the S0, S1.0, S2.0, and S3.0 nanocomposites. Indentation loads were introduced at different depths between 0 and 1000 nm. Figure 9a,c,e,g show the normal load against displacement (or penetration) diagram for S0, S1.0, S2.0, and S3.0, respectively. The penetration forces (load on the sample) for S0, S1.0, S2.0, and S3.0 were 23 mN, 21 mN, 26 mN, and 38 mN, respectively. It is evident that an increase in the CNT concentration in the compound enhances the normal load needed for penetration into the substrate. Figure 9b,d,f,h present the elastic modulus obtained by nano-indentation at various depths. The Young’s moduli of S0, S1.0, S2.0, and S3.0 were 31, 35, 45, and 49 GPa, respectively. It is worth noting that the elastic modulus can be attained quickly as a function of the depth. Three major factors affect the elastic modulus enhancement of S1.0, S2.0, and S3.0 composites: (a) load transfer efficiency at the HA/CNT interface, (b) higher modulus of elasticity of CNTs, and (c) uniform distribution of CNTs in the HA matrix^46,48^. Furthermore, the HA cations can react with the functionalized CNTs (carboxyl groups), possibly creating –COO–Ca–OOC– chemical bonding^49^. The nearby contact among the CNTs and the HA structure may create further CNT binding among the ceramic matrix, resulting in mechanical interlocking enhancement^47^. Therefore, the abovementioned mechanisms enhancing the bonding strength among the CNTs and HA causes an enhancement in the efficiency of the load transfer among the CNTs and HA matrix. It is thought that when composite material undergoes stress, first, the matrix deforms because of its lower modulus of elasticity. A proper stress transfer from the HA matrix to the CNTs and TiO_2_ may take place because of the sufficient bonding between the CNTs and the HA matrix at the interface. It has been suggested that TiO_2_ nanoparticles and mainly CNTs can absorb more energy and stress than hydroxyapatite, which leads to an enhancement in the modulus of elasticity of the HA/TiO_2_/CNT composite.

The actual elastic modulus may be affected by the concentration of CNT. The findings of this study indicate that the modulus of elasticity first rose as the indentation depth increased, then decreased to some degree after reaching 1000 nm in depth. Such a result could be attributed to the wear of the dense surface and densification of the inner layers, all of which occurred during the indentation. This result explains why the areas underneath the indenter stylus are denser, yet those on the other parts of the indenter are exposed to densification stresses and wear. Therefore, although the surface was penetrated by the tip of the indenter, the modulus of elasticity was enhanced because of the overall composite strength. Moreover, the densification of the region around the indentation is partly responsible for the increased material strength, which resulted in wear between the surfaces of the indenter and composite. The rule of hysteresis loop (residual stress) during loading–unloading suggests that the surfaces of composites encounter dissipation of elastic energy and achieve better stiffness, frictional energy, elastic response, and compressive plastic deformation. This dense area implies dissipated energy due to the plasticity of the material. Figure 9b–h (modulus of elasticity against displacement for S0, S1.0, S2.0, S3.0 nanocomposites) illustrates that Young’s modulus increased at shallow depths (0–400 nm), whereas it decreased from 400 to 600 nm. The Young’s modulus of S0 reached 31 GPa (at a depth of 230 nm) and declined as the depth of indentation increased. Besides, Young’s modulus of S1.0 was amplified until reaching 36 GP, after which it displayed a downturn as the indentation depth continued to increase. The elastic moduli of S2.0 and S3.0 increased to 45 GP and 49 GPa, then declined to 38 and 46 GPa, respectively.

**Figure 10:**
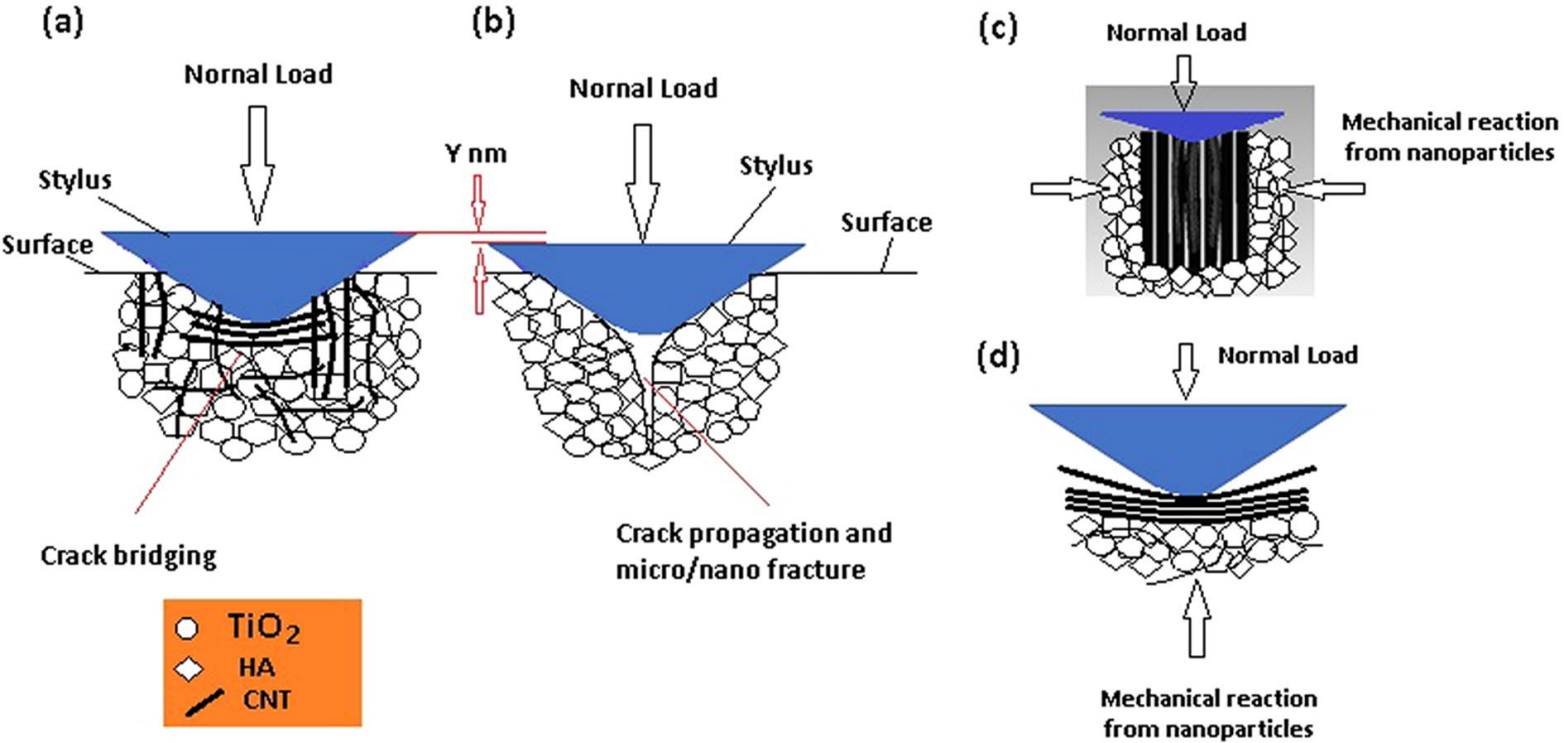
Schematic of nanoindentation and penetration of stylus under different conditions; indentation on (**a**) HA/TiO_2_ reinforced by CNTs, (**b**) HA/TiO_2_ reinforced, (**c**) vertical CNTs, and (**d**) horizontal CNTs.

With the absence of CNTs, the application of the normal load leads to compression of the HA and TiO_2_ nanoparticles, with the empty spaces between the particles getting smaller. In addition, owing to the weaker bonding between HA and the TiO_2_ nanoparticles, the stylus can easily separate them and penetrate to the substrate to greater depths (compared with HA/TiO_2_/CNT composite), the nano-/micro-cracks initiate and propagate with less resistance through the substrate and, therefore, create a larger stylus impression; consequently, less surface hardness would be achieved. However, CNTs prevent the easy separation of nanoparticles because of the bridging phenomenon, and they can also fill the empty spaces between the particles, resulting in less compression upon the application of the normal load. Moreover, the CNTs that are positioned vertically in the composites serve as columns that undergo buckling when the stylus surface pushes the tubes down. In contrast, the HA and TiO_2_ nanoparticles surrounding the CNTs (columns) serve as supports, holding the piles vertical and helping them resist the buckling, thereby, increasing the mechanical properties of the entire nanocomposite substrate. Figure 10a–d show the schematic of nanoindentation and penetration of the stylus under different conditions. Figure 10a shows that because of the existence of CNTs, the stylus penetrates a few nanometers (Y nm) less than the stylus shown in Fig. 10b; this can be due to the horizontal alignment of the CNTs, which undergo bending at the time of loading. These horizontally aligned tubes serve as bridges between the HA/TiO_2_ nanoparticles, resisting the cracks to be propagated. Furthermore, the nanoparticles surrounding the horizontal tubes, especially the ones at the bottom of the tubes, support the tubes, preventing bending under the normal load of the stylus (Fig. 10d). The vertically aligned nanotubes, which undergo buckling at the time of stylus penetration, are supports of the HA and TiO_2_ nanoparticles that surround the tubes and help the tubes resist against buckling (Fig. 10c).

### Tribomechanical properties

**Figure 11:**
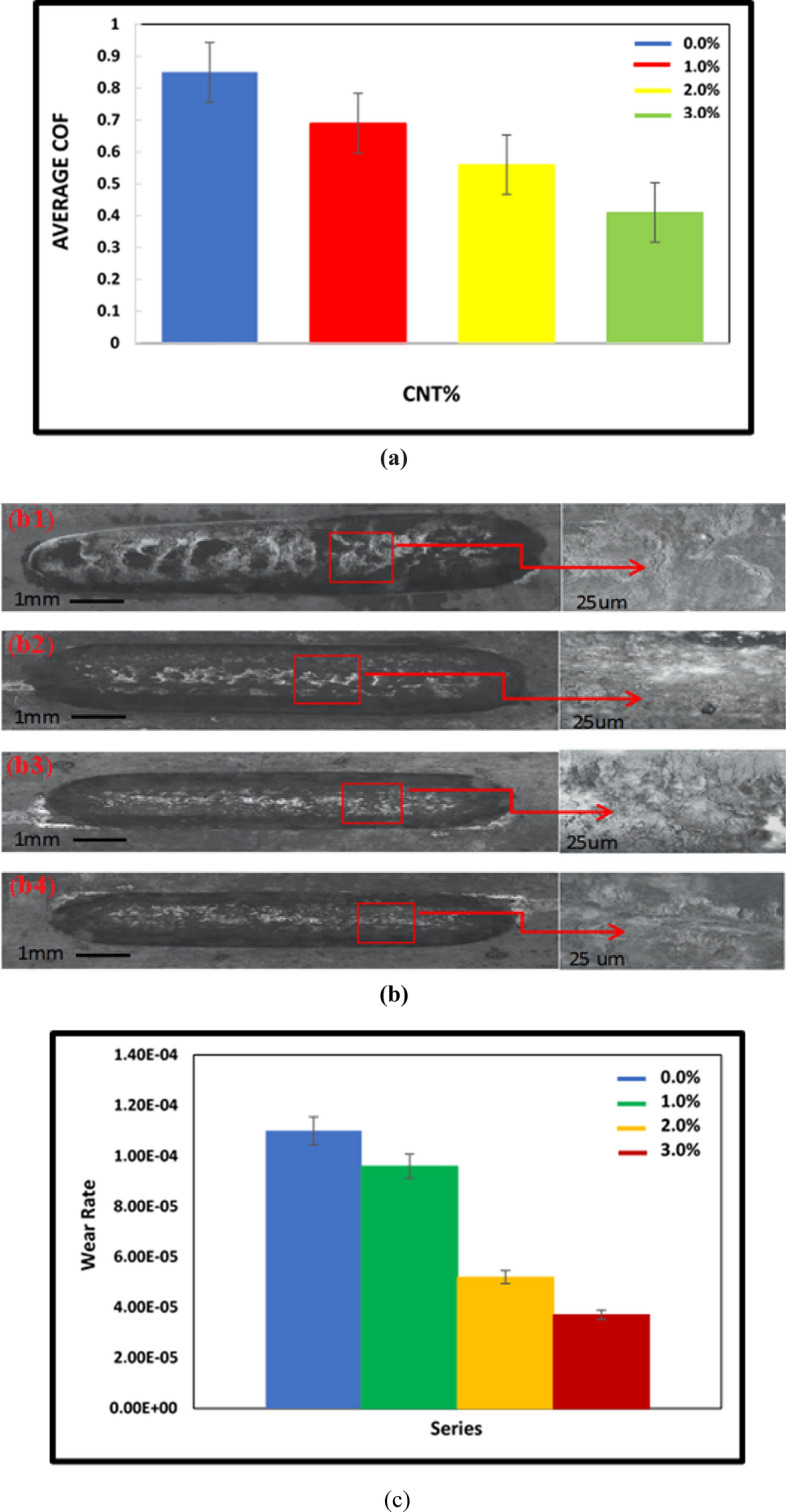
(**a**) Coefficient of friction of HA/TiO_2_/CNT 0%, 1.0%, 2.0%, and 3.0%, (**b**) Optical images of worn surface of the HA-TiO_2_-CNT samples with (b1) 0%, (b2) 1.0%, (b3) 2.0%, (b4) 3.0%, and (**c**) Effect of CNT content on the wear rate of HA/TiO_2_/CNT nanocomposites.

A reciprocating wear test was conducted to evaluate the tribological behavior of the nano-composites with various amounts of CNTs. The tribological behavior of the samples (S0, S1.0, S2.0, and S3.0) was calculated in terms of the coefficient of friction (COF) and wear mass loss. Wear mass loss is inversely proportional to the resistance to wear. The friction coefficients of the different samples as a function of CNT content are presented in Fig. 11a. It is evident that CNT has a significant impact on the coefficient of friction of the composites. The average friction coefficients for the S0, S1.0, S2.0, and S3.0 samples were 0.85, 0.69, 0.56, and 0.41, respectively. The S3.0 composite showed the lowest average friction coefficient.

Figure 11b (b1–b4) shows the top-view images of the different samples after wear testing. As the amount of CNT in the composite changed from 0% to 3.0%, the composite appeared to have greater resistance to wear than the S0 sample, owing to an improvement in the surface hardness. In addition, recent studies found that under physiological conditions, introducing HA into the coatings creates a lubricating layer with adequate resistance to wear on the surface. Phosphate anions in the composite are formed with hydrated ions and become an extensive hydration film that generates lubricated contact to reinforce the antifriction characteristic of the composite, like a molecular ball-bearing^50^.

A higher composite surface hardness leads to greater resistance to mass removal, thereby increasing the lateral (transverse) force. Increasing the lateral force while the normal force is kept constant leads to composites with higher COF.

Additional understanding of the wear characteristics of the S0, S1.0, S2.0, and S3.0 nanocomposites was obtained by investigating the morphology of the wear track. As shown in Fig. 11b1–b4, a flat morphology was observed in the wear trajectory of HA, which indicates the complete removal of the mass abrasive wear mechanism. The HA/TiO_2_ wear track showed displacement of mass toward the outer edge of the track, resulting in piling up effect. This is a specific characteristic of plastic deformation. While it is uncommon in brittle ceramics, such as TiO_2_ and HA, the reinforcement of CNTs in TiO_2_ and the HA matrix is the cause of this behavior in this case^51,52^. The wear tracks with higher magnification of different composites are also presented beside the corresponding figures. Figure 11 shows that the S0 composite with TiO_2_ particles suffered from intense wear with a rough and wide wear track. These characteristics suggest that the core wear mechanism of the S0 sample was abrasive wear^48,53^.

The debris was detected on the scratched surface, resulting from the delamination of the different nanoparticles in the reciprocating wear test. It was thought that the delamination was primarily attributed to the brittle fracture of the TiO_2_ sliding under the stainless steel ball. The high frictional shear stress due to the high COF was the reason for the brittle fracture between the stainless steel ball and the surface of the nanocomposite. Thus, brittle fracture and abrasive wear were the key mechanisms responsible for the wear scar of the S0 composite, resulting in a higher wear rate. From a comparison point of view, the width of the wear track in the S3.0 sample is smaller than those of the other samples because of its higher surface hardness and better lubrication (graphite removal from CNTs), which helped to withstand against the reciprocating ball and resulted in the removal of fewer particles and fragments.

Figure 11c shows the wear loss of composites under a 10 N normal load. The shear force of the wear probe may not be sufficient to scrape off the HA/TiO_2_ reinforced by CNT from the composite surface. The presence of CNT in S1.0, S2.0, and S3.0 decreases the wear mass loss of HA/TiO_2_ by 13.33, 66.67, and 53.33%, respectively. Because of its inhomogeneous microstructure and higher porosity, the wear loss of HA/TiO_2_ could be greater. The improved wear resistance of the CNT-containing composites is the product of their enhanced mechanical properties. The hardened CNT-reinforced composition prevents the loss of mass related to chipping and fracture during wear.

**Figure 12:**
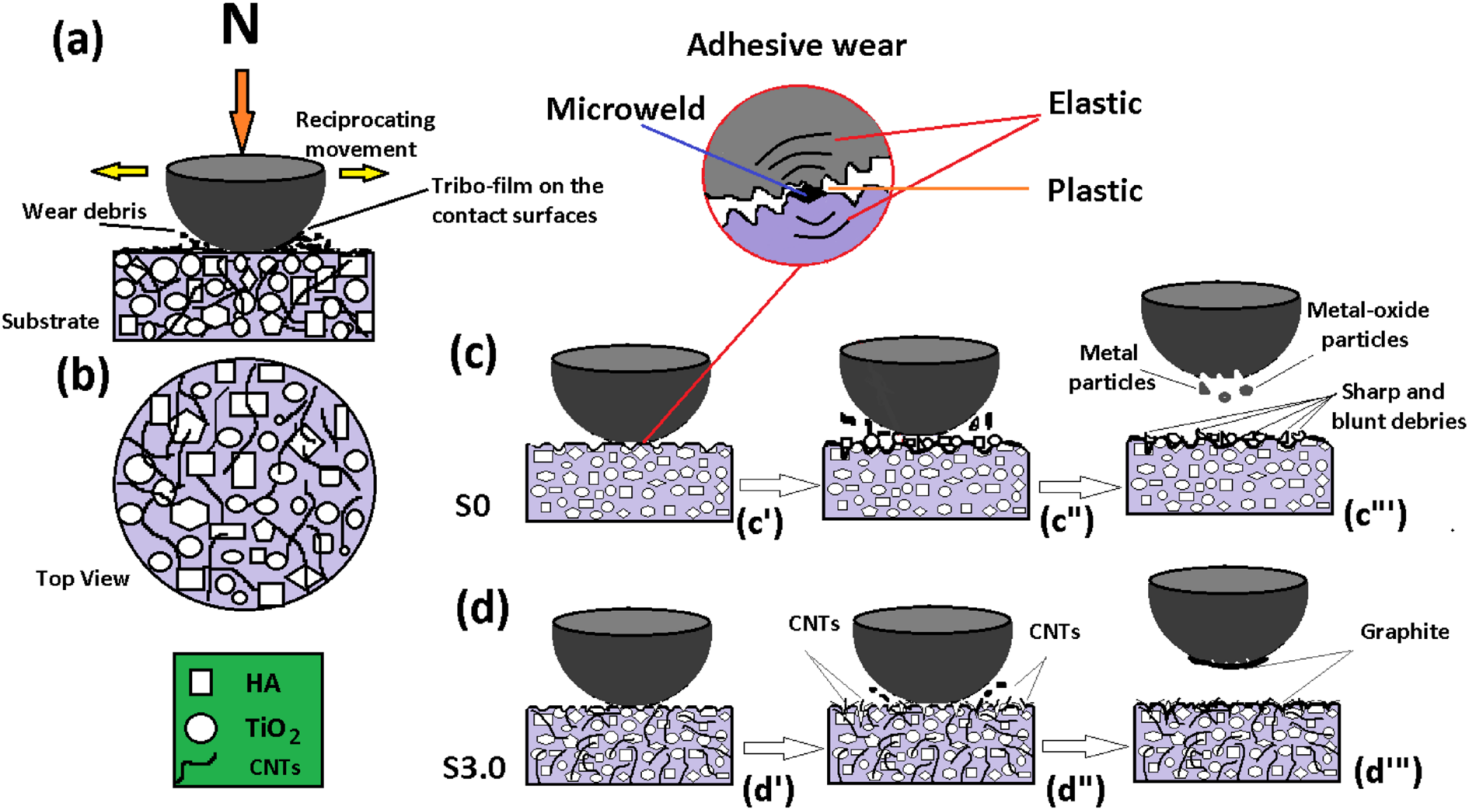
Schematic of (**a**) reciprocating wear, (**b**) top view of HA/TiO_2_/CNT nanocomposite, (**c**) reciprocating wear of S0 and (**d**) reciprocating wear of S3.0.

Figure 12a,b show a schematic of reciprocating wear and the top view of the tablet-shaped nanocomposite (contains HA, TiO_2_, and CNT particles). Figure 12a depicts the wear debris and the tribo-film on the contact surfaces due to the relative movement between the ball and substrate. Figure 12c (c′-c‴) shows a schematic of the wear process between the stainless steel ball and S0 sample. As the normal stress at these asperities is high, plastic deformation and adhesive bonds at the junctions (Fig. 12c′) are created. In addition, owing to the relative sliding between the stainless steel ball and nanocomposite substrate, surface films from both (ball and substrate) breakthrough (Fig. 12c″) and cause a temperature rise sufficiently to cause the migration/diffusion of atoms from one body to another body, resulting in the micro welding between asperities (Fig. 12c‴). Particles that are removed from one surface are either permanently or temporarily attached to the other surface. Owing to high temperatures and micro welding, the particles from the stainless steel are removed during the reciprocating movement. As the localized temperature is very high and oxygen is abundant, the metal debris is oxidized and creates hard metal-oxide particles. As the motion continues, these metal oxides, along with TiO_2_, contribute to an increase in the COF and the wear rate. Meanwhile, on the surfaces of the S3.0 sample and ball (lubricated surfaces because of the presence of graphite particles), the wear process is usually mild and generates fine and small debris and particles. Therefore, abrasive wear or delamination wear predominates under lubricated conditions, as graphite particles (removed from CNTs) serve as a coating of a solid lubricating system that separates the opposing surfaces while the lubricant itself wears away (Fig. 12d (d′–d″).

## Conclusion

In summary, nanocomposites of HA, TiO_2_, and CNTs were fabricated in situ by utilizing a combination of different techniques such as reverse microemulsion and the hydrothermal method. Furthermore, HIP was conducted at 1150 °C and 160 MPa as a consolidation procedure. CNT-containing composites exhibited enhanced biological and tribomechanical behavior in comparison with the behavior of the HA/TiO_2_ composite. A hardness test revealed that the surface hardness of the S1.0, S2.0, and S3.0 specimens improved by 116.8%, 129%, and 166.4%, respectively, over that of the S0 specimen. The results of the wear experiments showed that different rates of CNT can decrease the COF of composites. Moreover, the loss of mass due to wear in the S1.0, S2.0, and S3.0 specimens was greatly improved by 13.33%, 66.67%, and 53.33%, respectively, over that of the S0 specimen. The findings of the cell culture experiments suggest that adding CNT to the HA/TiO_2_ sample enhanced both cell proliferation and adhesion. Nanoindentation tests show that Young’s modulus of S0 reached 31 GPa and decreased as the depth of indentation increased. The Young’s modulus of S2.0 increased to 45 GPa, then decreased to 38 GPa. Likewise, for S3.0, the nano-hardness increased to 49 GPa at a depth of 500 nm and decreased thereafter.
